# Tuberculosis infection control in MDR-TB designated hospitals in Jiangsu Province, China

**DOI:** 10.1016/j.jctube.2025.100555

**Published:** 2025-07-29

**Authors:** Honghuan Song, Guoli Li, Zhuping Xu, Feixian Wang, Xiaoping Wang, Bing Dai, Xing Zhang, Jincheng Li, Yan Li, Limei Zhu

**Affiliations:** a Department of Chronic Communicable Disease, Jiangsu Provincial Center for Disease Control and Prevention, People's Republic of China; b Department of Chronic Communicable Disease, Wuxi Municipal Center for Disease Control and Prevention, People's Republic of China; c Tuberculosis Control Center, Suzhou Municipal Center for Disease Control and Prevention Tuberculosis Control Center, People's Republic of China; d Department of Chronic Communicable Disease, Nantong Municipal Center for Disease Control and Prevention, People's Republic of China; e Department of Chronic Communicable Disease, Zhenjiang Municipal Center for Disease Control and Prevention, People's Republic of China; f Department of Chronic Communicable Disease, Changzhou Municipal Center for Disease Control and Prevention, People's Republic of China; g Department of Chronic Communicable Disease, Yangzhou Municipal Center for Disease Control and Prevention, People's Republic of China

**Keywords:** Tuberculosis infection control, TB designated hospitals, TB prevention, High TB burden countries

## Abstract

**Background:**

Hospital-acquired Tuberculosis (TB) infections among healthcare workers (HCWs) and patients present a significant challenge due to the increased risk of TB infection within healthcare settings.

**Methods:**

A standardized assessment tool was applied for the evaluation, which involved direct observation, document review, and interviews with facility heads. A baseline evaluation of TB infection control (TBIC) measures in TB outpatient and inpatient departments, as well as laboratories, was completed by January 2019. Based on the results, a comprehensive intervention package was implemented, incorporating a three-tiered hierarchy of controls: administrative control (AC), environmental control (EC), and respiratory protection (RP). Subsequent monitoring was conducted quarterly, with corrective actions accordingly. More than two years of follow-up data were collected, with the collaboration of local hospitals, the municipality Centers for Disease Control and Prevention (CDC), and the Jiangsu Provincial CDC, concluding on August 31, 2021.

**Results:**

At baseline, the average implementation rates of AC, EC and RP were 57.3 %, 59.2 %, and 66.6 %, respectively. After the intervention, significant improvements were observed in key infection control measures. A triage process for cough patients was established, mechanical ventilation systems were installed, and the use of masks was improved. In addition, ultraviolet (UV) and upper-room ultraviolet germicidal irradiation (UVGI) systems were installed where required. As a result, the average implementation rates of AC, EC and RP significantly increased to 86.3 %, 87.4 %, and 98.4 % (P < 0.05), respectively. However, at the study’s conclusion, Suzhou Fifth People’s Hospital reported a lower AC implementation rate of 70.7 %, while Changzhou Third People’s Hospital had an EC implementation rate of 68.1 %. These discrepancies were primarily attributed to suboptimal architectural designs that hindered proper ventilation in the wards.

**Conclusions:**

This study demonstrates that designated hospitals still face persistent gaps in tuberculosis infection control (TBIC). However, over the course of one and a half years of targeted and standardized interventions, substantial improvements in TBIC practices were achieved across most participating institutions. Despite the suboptimal availability of dedicated TB wards, strengthening TBIC measures remains crucial to reducing TB transmission among healthcare workers and non-TB patients. This approach is both practical and scalable, particularly in high-burden TB settings. Nevertheless, the long-term efficacy and sustainability of these TBIC practices warrant ongoing evaluation.

## 1 Introduction

China continues to bear a high burden of tuberculosis (TB) [1]. Similar to other high-burden countries, hospital-acquired TB has long been recognized as an important infection risk for healthcare workers (HCWs) and other non-TB patients [[2], [3], [4]]. The increased risk of TB infection in healthcare settings is primarily attributed to occupational or hospital exposure, often due to undiagnosed or delayed diagnosis of TB, which results in TB infections among these groups [5]. Therefore, robust infection prevention and control (IPC) measures are needed to reduce the hospital-acquired TB risk.

HCWs are at a higher risk of acquiring TB compared to the general population, due to their possible occupational exposure to TB patients. Furthermore, TB transmission in hospitals not only heightens the risk for HCWs but also poses a threat to other patients and visitors .

Although the World Health Organization (WHO) and the China Centers for Disease Control and Prevention （CDC） have issued guidelines for TB infection control (TBIC) in healthcare settings, the limited availability of resources has hindered the full implementation of effective control measures, such as administrative controls (AC), environmental controls (EC) and respiratory protection (RP).

Jiangsu Province, located in eastern China, is one of the country’s leading regions in finance, education, technology, and tourism. In 2020, the TB incidence rate in Jiangsu was approximately 59 per 100,000, half of the national average [6], with 22,902 reported cases—an 8.97 % decrease from 25,159 in 2019. Every prefecture-level city in Jiangsu Province has set up a designated hospital for the diagnosis and treatment of drug-resistant TB.

In response to the need for better infection control, a comprehensive technical support package was introduced across 6 drug-resistant TB-designated hospitals in Jiangsu, including Suzhou Fifth People's Hospital, Wuxi Fifth People's Hospital, Changzhou Third People's Hospital, Yangzhou Third People's Hospital, Zhenjiang Third People's Hospital and Nantong Sixth People's Hospital, which are located in southern, Central, and Northern Jiangsu, respectively. The present study aimed to assess the implementation of TBIC measures before and after the introduction of this package.

## 2 Methods

### 2.1 Study design

The measurements used in this study were based on the TB comprehensive pilot project jointly carried out by Jiangsu CDC and China CDC, using the TB Building and Strengthening Infection Control Strategies (BASICS) program [7]. A set of standardized assessment tools was developed according to the WHO guidelines on tuberculosis prevention and control (2019update) [8], and the WHO recommendations for implementation of TBIC in healthcare settings. These tools covered various aspects of AC, EC, and RP, including 18, 16 and 6 independent test items, respectively. A before-after study was designed to assess improvements in the implementation of TBIC measures from October 2019 to September 2021. The study was conducted in the TB outpatient department, inpatient department, and laboratory of six drug-resistant TB-designated hospitals in Jiangsu province, China.

At each of the six institutional sites, an independent assessment team was established. Each team consisted of three members: one staff member from the municipal CDC, one from the hospital's infection control department, and one from the hospital's tuberculosis department. Prior to implementation, the Jiangsu CDC provided unified training for the six teams and played an active role in all baseline surveys. Subsequently, evaluations at each hospital were carried out by the corresponding municipal assessment teams, strictly following unified standards. The Jiangsu Provincial Center for Disease Control and Prevention was responsible for quality control and offered technical support throughout the assessment process. The study's start and end dates for each site were as follows: Suzhou Fifth People's Hospital (October 2019 to June 2021), Wuxi Fifth People's Hospital (October 2019 to July 2021), Changzhou Third People's Hospital (December 2019 to September 2021), Yangzhou Third People's Hospital (December 2019 to July 2021), Zhenjiang Third People's Hospital (December 2019 to July 2021), and Nantong Sixth People's Hospital (October 2019 to August 2021). Key information was collected and recorded at all sites. Based on the baseline assessment findings, a comprehensive intervention package was implemented, including AC, EC and RP following a three-level hierarchy of controls. After that, the assessment team conducted quarterly evaluations, supervised the execution of previous improvement plans, and formulated new improvement plans based on current assessment results. Continuous monitoring and improvements were made, resulting in a gradual increase in infection control levels. After the baseline assessment, six quarterly follow-up assessments were conducted, and endpoint assessment data were collected approximately one and a half years later, in collaboration with the hospitals, municipal CDC, and Jiangsu CDC. Due to the impact of the COVID-19 pandemic, the timeline for completing the assessment varied by institution.

## 3 Data management and statistical analysis

Data was collected in Excel tables and converted into scores. During the on-site evaluation of each indicator, three distinct scores were allocated: indicators fully implemented received a score of two points, those partially implemented were awarded one point, and those not implemented at all received zero points. The total score of each evaluation was the sum of the individual scores, with the maximum possible score being twice the number of indicators.

Implementation rate was calculated as follows:

Implementation rate =（Actual score / Maximum possible score) × 100 %.

Due to the varying applicability of indicators across different facilities and departments, the count of indicators differed among them. Proportions were calculated using SPSS version 23.0 (IBM Corp., Armonk, New York, USA).

China CDC suggested that implementation scores of 80 % or above were considered to meet the acceptable standards for Infection Prevention and Control protocols.

## 4 Results

### 4.1 The improvement of TBIC in the designated hospitals

**Fig. 1:**
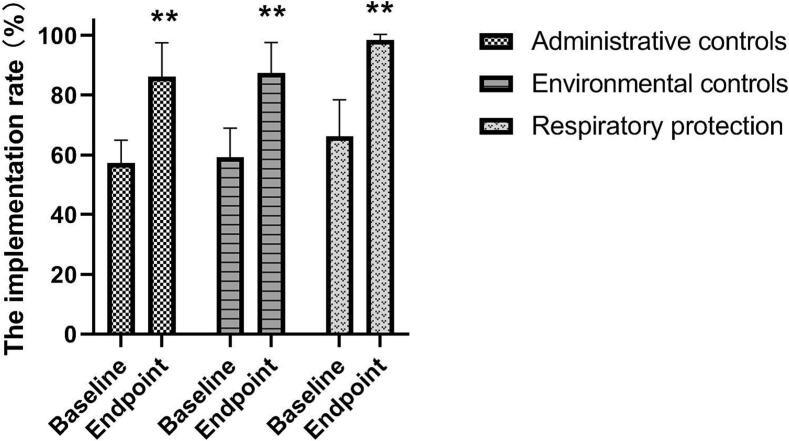
The improvement of TBIC in the designated hospitals. Data represents mean ± standard error of the mean. (**p < 0.01).

At baseline, the average implementation rates for AC, EC, and RP across the six hospitals were 57.3 %, 59.2 %, and 66.6 %, respectively. After the evaluation and implementation of interventions, a system for respiratory isolation and rapid triage of suspected tuberculosis patients was established in most hospitals. Mechanical ventilation, including the utilization of exhaust fans, was installed, and upper-room ultraviolet germicidal irradiation systems (UVGI) were deployed where required in outpatient departments and wards with insufficient ventilation. Additionally, a range of protective masks of various types and sizes were provided for medical staff, along with training on proper mask usage and fit testing to ensure adequate protection. As a result, the average implementation rates of AC, EC and RP significantly increased to 86.3 %, 87.4 % and 98.4 %, respectively (Fig. 1).

### 4.2 Improvement of TBIC in different hospitals

At baseline, the implementation rates for AC, EC and RP varied across the hospitals: 54.3 %, 69.6 %, 73.6 % in Suzhou Fifth People's Hospital; 70.8 %, 68.8 %, 75.7 % in Wuxi Fifth People's Hospital; 48.2 %, 44.9 %, 51.3 % in Changzhou Third People's Hospital; 58.2 %, 60.7 %, 74.7 % in Yangzhou Third People's Hospital; 53.5 %, 59.9 %, 49.7 % in Zhenjiang Third People's Hospital; and 58.2 %, 51.3 %, 72.3 % in Nantong Sixth People's Hospital, respectively.

**Fig. 2:**
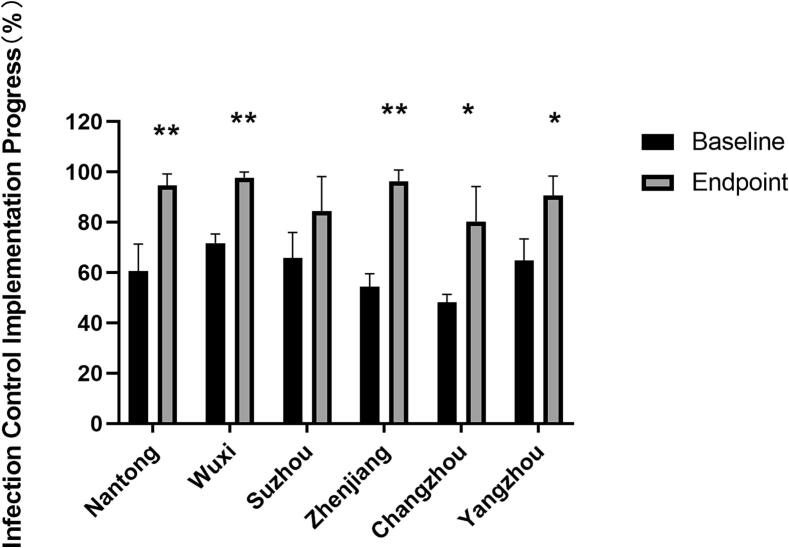
The improvement of TBIC in different hospitals. Data represents mean ± standard error of the mean. (**p < 0.01).

After six quarterly evaluations and subsequent implementation of interventions, the rates for AC, EC and RP showed significant improvements: 70.7 %, 84.4 %, 98.1 % in Suzhou Fifth People's Hospital; 97.6 %, 95.7 %, 100 % in Wuxi Fifth People's Hospital; 77.4 %, 68.1 %, 95.5 % in Changzhou Third People's Hospital; 82.1 %, 92.9 %, 96.9 % in Yangzhou Third People's Hospital; 97.7 %, 91.5 %, 100 % in Zhenjiang Third People's Hospital; and 92.1 %, 92.0 %, 100 % Nantong Sixth People's Hospital, respectively (Fig. 2).

At the endpoint, the implementation rate for AC in Suzhou Fifth People's Hospital and both AC and EC in Changzhou Third People's Hospital remained below 80 %.

### 4.3 Improvement of TBIC in hospital departments

At baseline, the implementation rates for AC, EC and RP in different departments were as follows: 49.9 %, 51.2 %, 60.8 % in the TB outpatient department; 59.4 %, 60.9 %, 69.5 % in the inpatient department; and 71.3 %, 65.4 %, 68.3 % in the laboratory, respectively.

**Fig. 3:**
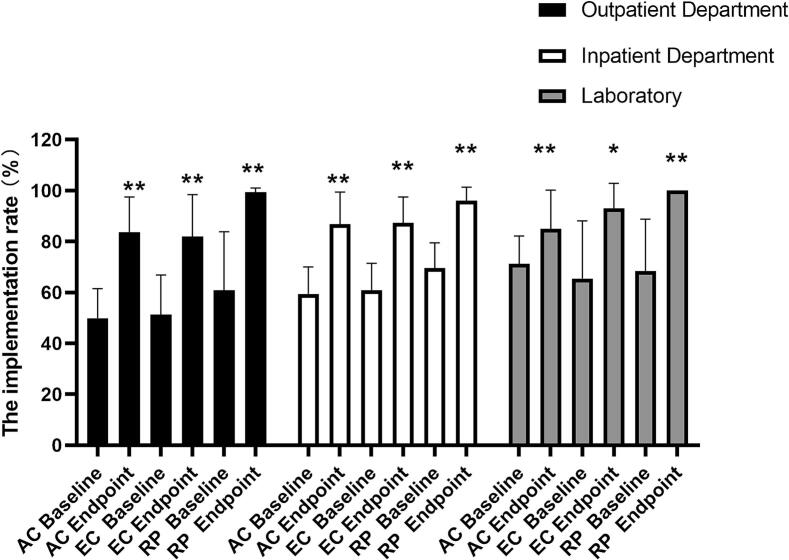
The improvement of TBIC in the departments of hospitals. Data represents mean ± standard error of the mean. (*p < 0.05, **p < 0.01).

After six quarterly evaluations and subsequent implementation of measures, the rates improved substantially: 83.5 %, 81.9 %, 99.3 % in the TB outpatient department; 86.9 %, 87.4 %, 96.0 % in the inpatient department; and 85.0 %, 93.0 %, 100 % in the laboratory, respectively (Fig. 3).

## 5 Analysis of main problems

**Fig. 4:**
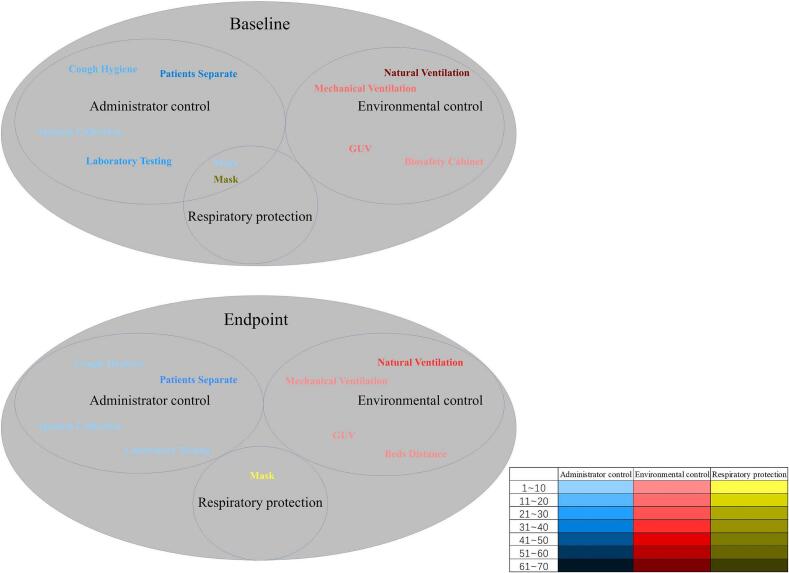
Analysis of the main problems at baseline and endpoint evaluation.

An analysis was conducted to identify the main issues faced by different departments at both the baseline and endpoint evaluation stages. The key problems identified in AC include the absence of patient triage protocols, insufficient education on cough hygiene, prolonged turnaround times for laboratory test results, and inadequate conditions for sputum collection. In terms of environmental controls EC, the main problems included a lack of ventilation management (both natural and mechanical), the absence of UV lights in some areas, and poor maintenance of biosafety cabinets. The main problem with RP was the lack of respirator training for healthcare workers. After 18 months of evaluation and implementation, although some problems persisted, significant improvements were observed compared to the baseline assessment. These improvements included the establishment of patients triage for those with coughs, the installation of mechanical ventilation, enhanced mask usage, the installation of UV lights where required, and the addition of the UVGI system in TB consultation rooms. (Fig. 4).

## 6 Discussion

TBIC is a key element in the global strategy to end the TB epidemic [9]. However, there are still critical infection control gaps in healthcare facilities, which continue to pose risk to HCWs, individuals living with HIV, and uninfected patients [[10], [11], [12]]. The widespread implementation of infection control measures, especially in settings with high caseloads, is essential to prevent further TB transmission and could ultimately serve as an important factor in the global TB epidemic [[13], [14]].

In China, as in many countries with high TB incidence and limited public − health resources, the implementation of TBIC measures has been insufficient [[15], [16]]. In Jiangsu Province, TB − designated hospitals, like those in most parts of the country, are also tasked with the treatment of respiratory − transmitted diseases such as COVID − 19 [17]. This co-designation underscores the need for better infection control: these hospitals treat both TB and COVID-19 patients, and inadequate infection control practices may result in patients from both groups developing secondary respiratory infections. Therefore, infection control is of particular importance. The aim of improving AC, EC, and RP is to reduce nosocomial transmission in these hospitals.

Due to the impact of the COVID − 19 pandemic and the differences in hospital resources, patient numbers, and the complexity of cases among different hospitals, the time required to complete the current study varies. For instance, hospitals with relatively limited resources faced challenges in rapidly recruiting participants, conducting tests, and analyzing data, which contributed to a prolonged study period of more than 18 months.

As a result, AC measures mainly focused on implementing existing policies. Key issues included the lack of triage for coughing patients, poor isolation of TB inpatient departments from other infectious diseases, insufficient patient hygiene education, and inadequate TBIC training for HCWs. A lack of knowledge, expertise, and experience in TB IPC, along with HCWs underestimating the airborne transmission risk, hindered the implementation of critical interventions. Limited expertise, funds for EC, and the absence of clear respirators reuse guidelines further elevated the risks of TB transmission between patients and HCWs. Our findings align with the research conducted by Paleckyte [18].

During this specific period, recommendations for domiciliary treatment included providing patients with separate, well-ventilated rooms and instructing them to wear masks. Patients were also advised to avoid crowded areas and public transportation. Moreover, family members were also trained in the fundamental TBIC knowledge and practices.

The deficiencies identified in the implementation of TBIC measures were addressed rapidly in most hospitals. EC issues mainly related to insufficient natural and mechanical ventilation in some consultation rooms and TB wards (<6 air changes per hour) and a shortage of UV lights. RP issues included inadequate protection for coughing patients and HCWs not carrying out fit tests for medical respirators. Despite these challenges, improvements were made at minimal cost. After 18 months of evaluation and implementation, a triage process for coughing patients was established, ventilation and masks usage were improved, UV lights were installed where required, and UVGI systems were added to TB consultation rooms and certain TB wards. The implementation rates for AC, EC and RP showed significant improvements in all hospitals. However, the main ongoing issue was the lack of a dedicated TB inpatient department, with separate TB wards remaining suboptimal in availability. A dedicated TB inpatient department is crucial because it effectively isolates tuberculosis bacteria from non-TB areas, preventing transmission to other patients, healthcare workers, and the public. Such isolation reduces the risk of new infections, thus protecting vulnerable populations.

At the AC level, the endpoint assessment results for all hospitals exceeded 80 %, except for Suzhou Fifth People's Hospital and Changzhou Third People's Hospital. In most hospitals, certain AC measures were relatively easy to implement, such as educating patients on cough etiquette, offering tissues or masks to visitors with cough symptoms, and setting up qualified sputum collection rooms. However, due to architectural layout constraints in the outpatient departments, Changzhou Third People's Hospital and Suzhou Fifth People's Hospital faced challenges with patient triage, making it difficult to effectively separate patients with suspected tuberculosis symptoms from others. Suzhou Fifth People's Hospital, which was burdened with a high volume of outpatients, also struggled with the laboratory's inability to report test results within 24 h (from the time of sputum collection to when the attending physician received the results).

Changzhou Third People's Hospital faced significant ventilation deficiencies in the outpatient and ward areas which were exacerbated by architectural limitations that were difficult to address. Despite the installation of UVGI devices, the implementation rate for EC remained low at 68.1 %.

The endpoint assessment indicated that the implementation rate for RP was the highest, with all six project sites exceeding 95 %. This is largely attributed to the provision of qualified protective masks and training for medical staff, both of which are relatively simple to implement.

Over the course of six quarterly assessments, the implementation rates for the all three infection control measures showed an upward trend. However, some regression was observed in some indicators. For example, increased patient volumes on specific days led to overcrowding in waiting rooms, and insufficient ventilation was noted in naturally ventilated wards or outpatient departmens during periods of low wind or calm weather conditions.

Overall, the implementation of IPC measures resulted in significantly improvements in hospital infection control, which is consistent with the findings of Canyou Zhang [7]. However, his research showed that the most substantial improvement occurred within the first two quarters. In contrast, our study experienced no significant improvements during the first two quarters, largely due to insufficient resources in the early stage and the outbreak of COVID − 19. Therefore, it is recommended that relevant departments provide more human and material support during the initial stages of IPC implementation.

## 7 Conclusions

The findings in this project demonstrate that significant gaps remain in TBIC within designated hospitals. Significantly, through focused and standardized efforts, substantial improvements in IPC practices were achieved across many institutions. To reduce the transmission of tuberculosis among HCWs and non-TB patients, it is essential to strengthen TBIC measures. This method is both practical and scalable, making it suitable for implementation in countries with a high burden of TB. However, the long-term impact and sustainability of the TBIC practices implemented should be carefully assessed.

## Ethics statements

This research is an indicator study conducted in hospitals and does not involve human or experimental animal subjects.

## CRediT authorship contribution statement

**Honghuan Song:** Writing – original draft, Formal analysis, Data curation, Conceptualization. **Guoli Li:** Software, Investigation, Data curation, Conceptualization. **Zhuping Xu:** Investigation, Data curation. **Feixian Wang:** Investigation, Data curation. **Xiaoping Wang:** Investigation, Data curation. **Bing Dai:** Investigation, Data curation. **Xing Zhang:** Investigation, Data curation. **Jincheng Li:** Investigation, Data curation. **Yan Li:** Writing – review & editing, Project administration, Methodology, Investigation. **Limei Zhu:** Visualization, Validation, Supervision, Software, Project administration.

## Funding

This work was supported by the Strengthening TBIC Management and Special Fund for Tuberculosis in Jiangsu Province. Additionally, it received funding from the Jiangsu Young Medical Talents Program (grant number: QNRC2016541) and the Jiangsu Provincial Medical Key Discipline Program (ZDXK202250).

## Declaration of competing interest

The authors declare that they have no known competing financial interests or personal relationships that could have appeared to influence the work reported in this paper.
